# Adiponectin in Cardiovascular Inflammation and Obesity

**DOI:** 10.4061/2011/376909

**Published:** 2011-09-15

**Authors:** Tamar R. Aprahamian, Flora Sam

**Affiliations:** ^1^Renal Section, Evans Department of Medicine, Boston University School of Medicine, Boston, MA 02118, USA; ^2^Cardiovascular Section, Evans Department of Medicine, Boston University School of Medicine, Boston, MA 02118, USA; ^3^Whitaker Cardiovascular Institute, Boston University School of Medicine, Boston, MA 02118, USA

## Abstract

Inflammation is widely known to play a key role in the development and progression of cardiovascular diseases. It is becoming increasingly evident that obesity is linked to many proinflammatory and obesity-associated cardiovascular conditions (e.g., metabolic syndrome, acute coronary syndrome, and congestive heart failure). It has been observed that adipokines play an increasingly large role in systemic and local inflammation. Therefore, adipose tissue may have a more important role than previously thought in the pathogenesis of several disease types. This review explores the recently described role of adiponectin as an immunomodulatory factor and how it intersects with the inflammation associated with both cardiovascular and autoimmune pathologies.

## 1. Adiponectin

Adiponectin is a cytokine, “adipokine”, produced almost exclusively in adipose tissue and is expressed at high levels by lean, healthy individuals. However, it has been reported that in pathological conditions such as coronary artery disease (CAD), diabetes mellitus, and hypertension that adiponectin levels decline [1–4]. The protein is present in human plasma at a range between 3 and 30 *μ*g/mL; however, as body mass increases, serum adiponectin levels decrease [5, 6]. The larger adipocytes found in obese subjects produce lower levels of adiponectin but higher levels of proinflammatory cytokines, such as TNF*α* [7]. The adiponectin monomer (30 kDa) has a structure consisting of a globular head and a collagenous tail, and this monomer is able to multimerize to form several stable complexes of low-, medium-, and high-molecular weight. Adiponectin shares sequence homology with collagens VIII and X as well as complement factor C1q. It has previously been referred to as ACRP30 for adipose complement-related peptide of 30 kDa based upon its homology to C1q [8]. In addition to promoting adipocyte differentiation, PPAR-*γ* agonists are known to increase adiponectin expression both in vitro and in vivo [9]. Adiponectin inhibits the expression of TNF-*α* in adipocytes, and both TNF-*α* and IL-6 inhibit the production of adiponectin [9, 10]. Negative regulation of adiponectin expression also results from hypoxia and oxidative stress [11, 12]. It is interesting to hypothesize that any or all of these factors could contribute to the vascular breakdown that could create a vicious cycle of perpetual inflammation in an obese state. 

Given that the levels of adiponectin vary in different inflammatory diseases as discussed above, these data suggest that the metabolic consequences observed in obesity may be related to an imbalance of pro- and anti-inflammatory cytokines.

Thus, adipokines contribute to the pathophysiology of obesity-linked disorders through their ability to modify proinflammatory and metabolic processes. Adipokines (such as leptin, TNF-*α*, PAI type 1, IL-1*β*, IL-6, and IL-8) are proinflammatory and increased in obesity [7]. In obese subjects, adiponectin levels are decreased, and the ability of adiponectin to inhibit the inflammatory processes is limited. Low adiponectin levels are inversely related to high levels of C-reactive protein (CRP) in patients with obesity, type 2 diabetes, and CAD [13–15].

Adiponectin also exerts anti-hypertrophic effects and protects against ischemia-reperfusion injury [16, 17]. Hypoadiponectinemia is a risk factor for patients with obesity-related complications such as CAD and hypertension [1–3, 18]. Hypoadiponectinemia also contributes to insulin resistance [19], impaired endothelium-dependent vasodilatation, impaired ischemia-induced neovascularization [20], salt-induced hypertension [21], and diastolic heart failure [22]. Thus, adiponectin mediates protective effects in obesity-related metabolic and vascular disease presumably by its anti-inflammatory actions and protects the heart against ischemia-reperfusion injury through its ability to suppress myocardial inflammation and apoptosis [23]. Similarly, lack of adiponectin exacerbates left ventricular hypertrophy and systolic heart failure and increases mortality after experimental aortic constriction [17, 24]. Hypoadiponectinemia appears to promote hypertension progression [2], but the mechanism by which this occurs is not entirely clear. 

Adiponectin supplementation suppresses the progression of viral myocarditis in diabetic obese mice [25], inhibits atherosclerosis progression in vitro by NF-*κ*B inhibition and phospho-Akt activation [26], and suppresses TNF*α*-induced I*κ*B*α* phosphorylation [27]. Interestingly, chronic adiponectin overexpression increased subcutaneous fat mass and protected against diet-induced insulin resistance [28].

## 2. Shifting Paradigm of Adiponectin Expression in Disease

Obesity is considered a state of mild inflammation, and it is well documented that increasing adiposity results in a decrease in adiponectin production, thus, perpetuating an inflammatory state. This occurs in patients with metabolic syndrome, type II diabetes, and cardiovascular disease. In addition, low plasma adiponectin has been associated with myocardial infarction in young patients independent of other conventional risk factors [29]. Of interest, in diseases that are not necessarily related to obesity, adiponectin levels have recently been shown to be increased in chronic inflammatory and autoimmune diseases such as type I diabetes, SLE, rheumatoid arthritis, inflammatory bowel disease, and chronic systolic heart failure. The latter is contrary to the decrease in adiponectin levels in heart failure related to obesity [30]. Adiponectin levels are also increased in human patients with hypertrophic cardiomyopathy associated with diastolic dysfunction [31]. 

**Figure 1:**
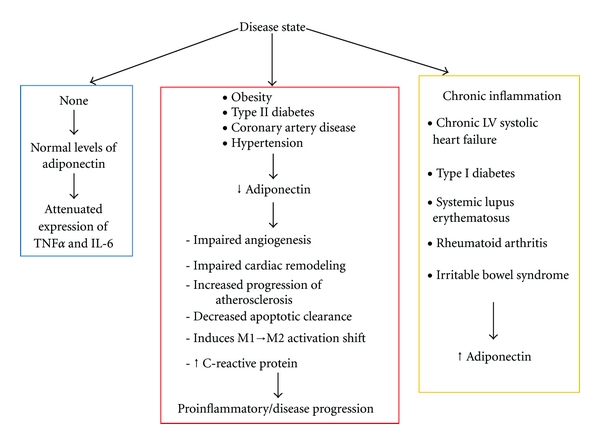
Adiponectin levels and inflammatory state. In healthy individuals, adiponectin maintains anti-inflammatory properties. Disease states where adiponectin levels decrease result in proinflammatory signaling and exacerbation of disease. Recent data has shown that adiponectin levels are increased in chronic inflammatory diseases, but the reason for this is incompletely understood.

In this review, we will discuss the various conditions in which levels of adiponectin are known to be dysregulated and the impact that these scenarios of inflammation have on disease progression, in particular, the impact on the cardiovascular system (Figure 1).

## 3. Cardiac Remodeling, Hypertension, and Heart Failure

Hypertension is associated with left ventricular hypertrophy which can eventually lead to heart failure. In a mouse model of transverse aortic constriction, adiponectin deficiency results in concentric left ventricular hypertrophy and greater mortality at a short-time point; however, when the time course is extended, the results are limited to the prevention of left ventricular remodeling and preserved mitochondrial oxidative capacity [17, 32]. Consistent with this remodeling data, pioglitazone—a known modulator of inflamation and an activator or adiponectin—reduced cardiac hypertrophy and fibrosis in wild-type mice subjected to angiotensin-II-infusion. These beneficial effects were lost when the same injury and treatment were performed on adiponectin-deficient mice [33]. When adiponectin-deficient mice are maintained on a high-fat/high-sucrose/high-salt diet, the mice become obese, insulin resistant, and hypertensive [34]. In the KKAy mouse, a murine model to study metabolic syndrome [35, 36], adiponectin administration improved hypertension [21]. Antihypertensive drugs were used to determine adiponectin production in vitro, and it was found that there are varying degrees of adiponectin secretion which may impact the results of the drug efficacy [37]. With regards to human studies, significantly lower levels of adiponectin have been found in patients with essential hypertension compared to normotensive controls [38]. Taken together, these data suggest that adiponectin plays an important protective role in the development of hypertension. 

While these animal models of heart failure have shown that adiponectin protects against the development of systolic dysfunction, adiponectin levels are increased in humans and may be predictive for mortality, in patients with chronic heart failure [39]. The exact cause of the upregulation is unknown, but several hypotheses are likely. First, it is possible that increased expression of adiponectin may be a compensatory response to the stress of heart failure, similar to the mechanism described for B-type natriuretic peptide (BNP) secretion [40]. Although the molecular mechanisms are unknown, BNP levels correlate with adiponectin levels in human heart failure [39, 40]. Furthermore, direct stimulation of human adipocytes with BNP has been shown to result in a cGMP-dependent release of adiponectin [41]. A second possibility for elevated adiponectin levels in cardiac disease could be the development of a condition termed “adiponectin resistance.” Although the mechanism has not been elucidated, adiponectin resistance has been described in a small number of published studies in both human tissue and animal models [42–44]. Furthermore, as observed in systolic heart failure, increased levels of adiponectin may result as a compensatory response to aberrant expression of adiponectin receptors. Therefore, disrupted adiponectin signaling in target tissues (resulting from a change in adiponectin receptor expression) may act as a compensatory response and partially explain the observed increase in adiponectin levels in these disease states.

## 4. Coronary Artery Disease

The relationship between adiponectin levels and coronary artery disease and acute coronary syndrome is not so straightforward. There is an inverse relationship between serum adiponectin levels and nondiabetic patients with regard to the severity of coronary artery disease. In type I diabetic patients and nondiabetic controls, hypoadiponectinemia is associated with coronary artery calcification [45]. In addition, adiponectin levels are significantly lower in patients with acute coronary syndrome compared to patients with stable angina and healthy controls [46]. Furthermore, the development of atherosclerosis and coronary artery disease goes hand in hand. Adiponectin has anti-atherosclerotic, as well as anti-inflammatory properties that may play an important role in preventing the progression of coronary artery disease. Results from clinical surveys show that low adiponectin levels, while being a predictive marker for early-stage atherosclerosis, are also significantly associated with coronary artery disease [3].

## 5. Accelerated Atherosclerosis and Lupus

Accelerated atherosclerosis is believed to be a critical factor contributing to stroke and coronary heart disease (CHD), a leading cause of death among young women with SLE. Clinical studies have largely examined the relation between SLE and endpoint cardiac events including myocardial infarction and stroke [47, 48]. More recently, attention has shifted towards the causes of advanced cardiovascular diseases, the focus now being on the contribution of accelerated atherosclerosis in SLE patients. To complicate matters, it has been shown that there is an increased incidence of accelerated atherosclerosis, and as a result, increased endpoint cardiac events in patients with SLE. The exact cause is unknown, but chronic inflammation is likely a contributing factor. 

Adiponectin plays a role in the inflammatory components of cardiovascular diseases. To begin with, lack of adiponectin in a mouse model of atherosclerosis leads to not only an increase in T-cell accumulation within the lesions, but also an increase in total atherosclerotic lesion area compared to adiponectin-sufficient controls [49]. Adiponectin is also known to be upregulated by PPAR*γ* and treatment with PPAR*γ* agonists results in decreased atherosclerosis [50]. A meta-analysis of studies to determine the renal protective effects of PPAR*γ* agonists on diabetic patients showed that urine albumin excretion was decreased in patients receiving PPAR*γ* agonists [51]. Based on this information, our lab hypothesized that the several immunomodulatory effects of PPAR*γ* agonists could potentially be beneficial in lupus since glomerulonephritis is a major complication of lupus. Inducing adiponectin upregulation by maintaining two different mouse models on a PPAR*γ* agonist-containing diet resulted in significantly less disease than mice receiving vehicle treatment. We further showed that these effects were mediated by adiponectin [52]. These data suggest that the increased level of adiponectin derived from upregulation by PPAR*γ* plays a major role in the decrease of inflammation in these models. 

Other studies have utilized mouse models to further elucidate the interaction between atherosclerosis and lupus. Several different lupus-like mouse models, including *gld*, *lpr*, and *Sle*1.2.3, have been used in the generation of various chimeras with either apoE−/− or LDLr−/− mice, which are prone to atherosclerosis. These models consistently report that lupus disease and vascular complications are worsened when simultaneously present, with inflammation likely being a contributing factor [53–56]. The involvement of adiponectin has not been examined in these models; however, given the importance of this adipokine in regulating SLE as well as inflammatory processes involved in atherosclerosis, it would be of great interest to study not only the levels of adiponectin, but also to administer exogenous adiponectin to determine if the phenotypes could be rescued. 

## 6. Pulmonary Disease and Pulmonary Hypertension

Cardiopulmonary complications are widely recognized causes of morbidity and mortality especially in obese adults [57]. Several instances have been described where adiponectin (or lack thereof) is involved in the progression of the disease. The presence of adiponectin in lung was first described by Summer et al. In this study, the authors show that alveolar macrophages from mice lacking adiponectin spontaneously produce increased levels of TNF*α* in vitro. In addition, these mice have increased expression of proinflammatory cytokines and matrix metalloproteinases, and the lungs display an emphysema-like phenotype including dilated air spaces, decreased tissue elastance, and increased lung displacement volume [58]. The same group has shown that adiponectin is found on the luminal side of the lung blood vessels and serves to inhibit TNF*α*-induced upregulation of E-selectin which is expressed when endothelial cells become inflamed. There was also evidence of pulmonary hypertension associated with the infiltration of perivascular inflammatory cells in an adiponectin-deficient mouse model [59]. In a similar vein, allergic airway inflammation, a model of chronic asthma, induced in adiponectin-deficient mice resulted in pulmonary vascular remodeling and pulmonary arterial hypertension [60]. Overexpression of adiponectin has direct effects on pulmonary artery smooth muscle cells in a murine model of inflammation-induced pulmonary hypertension. The result was reduced disease and improved pulmonary arterial remodeling [61]. Similarly, hypoxia-induced pulmonary arterial remodeling and right ventricular hypertrophy can be attenuated by overexpression of adiponectin [62]. More recently, it has been shown that patients with higher levels of plasma adiponectin after acute respiratory failure have a higher risk of mortality. Analysis of serum adiponectin at day 1 or day 6 after respiratory failure showed that low levels of adiponectin at the earlier timepoint were associated with increased survival [63]. Further study is required to explain the different results observed between human and animal studies. It is possible that, in humans with an acute illness, the increased levels of adiponectin could be a compensatory response or could be a result of resistance with relation to adiponectin receptors. Taken together, the mouse and human data suggest that adiponectin plays a role in pulmonary disorders and may play a role in modulating the acute response to critically ill patients, which is an area of research that should be further studied.

## 7. Angiogenesis

Prior data from our lab and others has shown that adiponectin modulates angiogenesis in vitro and in vivo. This has been demonstrated using mouse models of tissue ischemia and cardiac remodeling as well as rabbit corneal models of angiogenesis [20, 64, 65]. For example, pre-treatment with adiponectin of endothelial progenitor cells for angiogenic cell therapy results in enhanced survival and proliferation of the cells in an ischemic hind-limb model, suggesting that high levels of adiponectin are essential for a robust angiogenic response [66]. Furthermore, adiponectin-deficient mice have a poor angiogenic response to ischemic hind-limb surgery; however, the systemic administration of adiponectin restores the angiogenic response to that seen in wild-type controls [20]. A mouse model of cardiac specific angiogenesis, transverse aortic constriction was performed in both adiponectin-deficient and wild-type mice, and a reduction in capillary density was observed as well as an increase in LVH, pulmonary congestion, and a reduction in LV systolic function in the mice lacking adiponectin. Once again, these data suggest that adiponectin is involved in angiogenesis related to cardiac remodeling [65]. One mechanistic explanation for the beneficial effects of adiponectin in angiogenesis has been demonstrated to occur through AMPK-dependent regulation. Another branch of the angiogenic regulatory pathway is implicated by in vitro and in vivo data linking adiponectin to a positive revascularization response to ischemia via COX-2 signaling which is known to be vasculo-protective [67]. Adiponectin supplementation to wild-type mice also resulted in an increased rate of recovery with regard to limb perfusion. Thus, adiponectin is able to act in several ways to promote a favorable angiogenic response. 

## 8. Obesity, Vascularity, and Angiogenesis

Given its relationship to cardiovascular complications and the clinical importance of obesity in the United States, it is somewhat surprising that only recently has the importance of adipose tissue microenvironment been addressed in molecular studies related to metabolic dysfunction. Both animal models and obese human studies demonstrate that total fat mass does not necessarily provide an indicator for metabolic status. These types of studies have led to the thought that metabolic dysfunction is not related to the adipose tissue *quantity* alone, but rather, the *quality* of the individual adipocyte [28]. The factors contributing to the characteristics of the adipocyte in this context are likely related to the status of fat pad inflammation and its perfusion by the microvessels. It is possible that reducing the blood flow in the adipose tissue may result in spontaneous necrosis of the large adipocytes that are present in obese states, and this will contribute to inflammation. Numerous studies have shown that adiponectin deficiency leads to diminished tissue perfusion and that elevated adiponectin levels promote vascular growth in skeletal muscle and tumor vascularity. However, to date, there are no studies that have examined the role of adiponectin in adipose tissue perfusion. Capillary rarefaction in the fat pads has been observed in obese mice, and this has also been correlated with a decrease in the expression of the angiogenic growth factor VEGF, which can lead to adipose tissue hypoxia [68, 69]. This blood flow restriction in the adipose tissue could contribute to the propagation of the inflammation. Recent studies have begun to determine the impact of fat pad expansion on adipose tissue hypoxia and capillary density in mouse models and humans. It has been suggested that spontaneous necrosis will occur in larger-sized adipocytes as a result of the limited oxygen from the circulation. Thus, there is a recruitment of macrophages to the adipose tissue in order to phagocytose the dying cells. It is possible that adiponectin, since it has a major role in both cardio-protective and anti-inflammatory processes, also plays a role in adipose tissue vascularity and inflammation.

## 9. Macrophage Phenotype and Obesity

Macrophages found in the adipose tissue contribute greatly to obesity-related metabolic dysfunction and chronic inflammation [69]. A correlation has been made in both humans and animal models showing that macrophage infiltration leads to the development of insulin resistance [12, 70, 71]. The accumulation of “crown-like” structures, which are markers of “inflamed fat”, suggests that clearance of dead adipocytes by macrophages is impaired, and this may be due to dysregulated adipokine levels or another obesity-related factor [72]. 

Macrophages can be characterized as M1 or M2 depending on their activation phenotype, which is similar to Th1/Th2 polarization. “Classically activated” macrophages are referred to as having an M1 phenotype and can upregulate cytokines generally involved in pro-inflammatory processes such as TNF*α*, IL-6, and IL-12. In addition, M1-type macrophages can increase the production of reactive oxygen species and nitrogen intermediates. “Alternatively activated” macrophages are categorized as having an M2 phenotype. These macrophages secrete IL-10, which is an anti-inflammatory cytokine and partake mostly in the downregulation of pro-inflammatory cytokines. M2 macrophages can also upregulate arginase-1 which has the ability to diminish the inducible nitric oxide synthase reaction. Other functions of M2 macrophages include the ability to upregulate scavenger receptors, mannose receptors, and the IL-1 receptor antagonist [73–75]. Characterization of macrophages from adipose tissue shows differential activation phenotypes dependent on obesity. Diet-induced obesity in a mouse model results in adipose tissue macrophages that have an M1-type phenotype. However, in lean mice, macrophages express markers of the M2 phenotype suggesting a switch from an anti-inflammatory phenotype to a more pro-inflammatory phenotype [76]. Therefore, it is reasonable to suggest that diet-induced obesity is capable of shifting the activation characteristics of macrophages from the protective M2-phenotype, to the pro-inflammatory state leading to metabolic dysfunction characterized by the M1-phenotype. 

Analysis of peritoneal macrophages and the stromal vascular fraction of cells from adipose tissue of adiponectin-deficient mice reveals a shift towards increased expression of cytokines related to macrophages with an M1-type pro-inflammatory phenotype. In contrast, exogenous overexpression of adiponectin can decrease the generation of reactive oxygen species, while also shifting the macrophage population to those of an M2-type phenotype [77].

Taken together, it is interesting to speculate that the power of adiponectin in shifting inflammatory properties of macrophages within adipose tissue combined with the data that adiponectin-deficient mice are unable to adequately respond to an ischemic event may be important in the role of the microenvironment of adipose tissue with relation to metabolic consequences and the progression of cardiovascular diseases. 

## 10. Apoptotic Cell Clearance

Adiponectin is structurally similar to complement C1q, which is known for its role in the complement cascade and its ability to bind apoptotic bodies and facilitate their removal via a non-inflammatory process of phagocytosis by macrophage. C1q belongs to the family of collectin proteins and among their many anti-inflammatory functions, a major role is to facilitate clearance of apoptotic cells, thus, maintaining a quiescent, anti-inflammatory state. It was shown in vitro that adiponectin can similarly facilitate the clearance of apoptotic bodies via calreticulin receptor on macrophages, resulting in a “quiet” phagocytosis [78]. To further corroborate these findings in vivo [79], exacerbation of the lupus phenotype was observed in a lupus-prone mouse model lacking adiponectin, and this was shown to be partially due to impaired clearance of apoptotic material. Taken together, these data provide evidence that adiponectin can facilitate a non-inflammatory removal of apoptotic bodies. To extend these findings to another pathological condition, serum adiponectin levels are reported to decrease in patients with early loosening of hip replacement [80]. The authors hypothesize that decreased clearance of apoptotic cell remnants due to low adiponectin may contribute to the degeneration of the hip replacement; however, this is an area that is currently under investigation. These data provide further evidence that adiponectin could play a major role in both apoptotic cell clearance as well as bone destruction/creation. 

With respect to obesity, the observation of “crown-like” structures (CLS) in adipose tissue is evidence of pro-inflammatory occurrences in obese subjects and animals [72, 81]. These CLS are rings of macrophages usually surrounding an adipocyte and could be the result of a lack of anti-inflammatory adipokines or the inability of macrophages to clear dead adipocytes, both of which are associated with obesity. Under normal conditions, clearance of early apoptotic cells is a non-inflammatory process. However, under obese conditions, the constant accumulation of dying adipocytes could be sensed by the macrophages to promote a proinflammatory response and, therefore, aggravate the inflammation. This impaired clearance of apoptotic cells in obesity is of particular interest since the lack of adiponectin could potentially play a role in driving inflammation resulting from the accumulation of CLS in obesity-related inflammation.

## 11. Concluding Statements

Adiponectin is a highly circulating adipokine that maintains its anti-inflammatory protective effects. Research continues to show its diverse functions in various disease states. It will be imperative to clarify the role of adiponectin in cardiovascular disease with relation to inflammation. This is complicated by the fact that low levels of adiponectin occur in obesity, type II diabetes, and metabolic disorders, whereas high levels of adiponectin are found in heart failure and hypertension, as well as chronic inflammatory autoimmune diseases such as SLE, type I diabetes, and rheumatoid arthritis.
